# Combined metabolic-reproductive association and predictive value of AMH and TyG index in PCOS: a single-center retrospective study

**DOI:** 10.3389/fendo.2026.1847801

**Published:** 2026-07-08

**Authors:** Yu Chen, Xinyan Shi, Lin Ma, Yujie Hu, Mei Wang, Lei Shu

**Affiliations:** 1 Department of Clinical Laboratory, Hangzhou Women’s Hospital, Hangzhou, Zhejiang, China; 2 Department of Reproductive Medicine and Endocrinology, Hangzhou Women’s Hospital, Hangzhou, Zhejiang, China

**Keywords:** anti-Müllerian hormone, insulin resistance, polycystic ovary syndrome, predictive value, triglyceride-glucose index

## Abstract

**Background:**

Polycystic ovary syndrome (PCOS) is a heterogeneous endocrine disorder associated with both reproductive and metabolic dysregulation. While anti-Müllerian hormone (AMH) and the triglyceride-glucose (TyG) index have been independently linked to PCOS, their combined and interactive effects remain unclear. This study aimed primarily to investigate the independent and synergistic associations of AMH and the TyG index with PCOS, and secondarily to evaluate their predictive performance for PCOS risk.

**Methods:**

A single-center retrospective cross-sectional study included 628 participants (2020-2024). Clinical, hormonal, and metabolic parameters were assessed. Associations and predictive ability were examined using logistic regression restricted cubic splines (RCS), subgroup analysis, and receiver operating characteristic (ROC) curves.

**Results:**

Adjusted analyses confirmed AMH and the TyG index as independent risk factors for PCOS (*ORs* = 1.252, 1.981; both *P* < 0.05). A significant interaction was identified (*OR* = 1.029, *P* < 0.001). In subgroup analyses, a significant positive association was observed between the TyG index and PCOS risk in the low AMH subgroup (*OR* = 4.561, *P* = 0.002). Compared with the double-low group (low AMH and low TyG), the risk for PCOS showed a sequential increase in the low AMH/high TyG, high AMH/low TyG, and double-high groups (*ORs* = 3.582, 5.894, and 12.082; all *P* < 0.05), demonstrating a clear dose-response relationship. The predictive model combining AMH and the TyG index demonstrated the highest accuracy (AUC = 0.693), which was slightly higher than that of either indicator alone.

**Conclusion:**

Beyond their independent roles, AMH and the TyG index interact synergistically in the pathogenesis of PCOS.

## Introduction

Polycystic Ovary Syndrome (PCOS) is a prevalent endocrine and metabolic disorder, affecting approximately 6% to 22% of women of reproductive age (1, 2). Its clinical presentation is highly heterogeneous, encompassing core features of reproductive endocrine abnormalities—such as hyperandrogenemia, ovulatory dysfunction, and polycystic ovarian morphology—and significant metabolic disturbances, with insulin resistance (IR) being central (3–5). A growing body of evidence indicates that this comorbidity of metabolic and reproductive dysfunction signifies that PCOS is not a single disease entity but rather a spectrum disorder comprising distinct dominant phenotypes (6–9). This marked heterogeneity results in considerable variation among patients in clinical manifestations, therapeutic responses, and risks of long-term complications, including type 2 diabetes, cardiovascular disease, and infertility (10, 11). Consequently, a key challenge in optimizing the clinical management of PCOS lies in refining and supplementing the current diagnostic criteria to develop novel tools capable of accurately identifying high-risk subtypes and enabling individualized risk prediction and management.

The pathophysiology of PCOS is complex and reflects interactions among genetic, metabolic, fetal, and environmental factors, the relative importance of which may vary among individual affected women. Prominent features include disordered gonadotropin secretion, hyperandrogenemia, insulin resistance with compensatory hyperinsulinemia, ovarian dysfunction, and follicular arrest (12). On the one hand, dysfunction of the hypothalamic-pituitary-ovarian (HPO) axis leads to abnormalities in the pulse frequency and amplitude of luteinizing hormone (LH), which in turn triggers ovarian-derived androgen oversecretion and ovulatory dysfunction (13, 14). Anti-Müllerian hormone (AMH), a key hormone secreted by granulosa cells, is significantly elevated in the serum of PCOS patients (up to 2–3 times that of normal women) (15) and is regarded as a sensitive clinical marker reflecting the preantral follicle pool reserve and polycystic ovarian morphology (16). Emerging research further suggests that elevated AMH may play a significant role in the neuroendocrine dysregulation of PCOS by directly or indirectly modulating the activity of hypothalamic gonadotropin-releasing hormone (GnRH) neurons (17). On the other hand, insulin resistance of varying degrees accompanied by compensatory hyperinsulinemia is present in up to 50–70% of PCOS patients, constituting the core of its metabolic disturbances (18, 19). Hyperinsulinemia can synergize with LH to stimulate ovarian theca cells to produce more androgens. Conversely, the hyperandrogenic environment can exacerbate insulin resistance in peripheral tissues (e.g., muscle, adipose tissue) (20), thereby establishing a self-reinforcing vicious cycle. This mutual exacerbation between the metabolic and reproductive axes forms the pathological basis for disease progression and long-term health risks in PCOS.

However, current clinical management of PCOS faces certain limitations and challenges in risk assessment and stratification (21). The Rotterdam diagnostic criteria, widely adopted internationally (3), are primarily designed for disease identification rather than for the quantitative stratification of disease severity, subtype classification, or long-term risk. Although studies have identified several potential biomarkers, including AMH, lipid profiles, and insulin resistance-related indicators, there remains a lack of an integrated, easily applicable risk stratification system in clinical practice that effectively combines information from both reproductive and metabolic dimensions. The triglyceride-glucose (TyG) index, a simple parameter calculated from routine biochemical measures (fasting triglycerides and glucose), has been extensively validated as a reliable surrogate marker for assessing insulin resistance and cardiometabolic risk (22, 23). It has also shown potential for association with disease phenotypes and adverse reproductive outcomes in the PCOS population (24, 25). AMH and the TyG index reflect the pathophysiological state of PCOS from two core dimensions: “ovarian reserve” and “metabolic homeostasis,” respectively. However, whether a synergistic effect exists between them in risk prediction, and whether a combined strategy can provide superior stratification efficacy, have not yet been systematically validated through model construction.

Based on the above background, this study has two main goals. The primary objective is to investigate the independent and interactive associations of AMH and the TyG index with PCOS using cross-sectional data. The secondary objective is to explore the potential of a combined stratification approach, with the aim of generating hypotheses and providing preliminary evidence for future research.

## Materials and methods

### Study design

This was a single-center retrospective cross-sectional study conducted at the Reproductive Endocrinology & Infertility and General Gynecology clinics of Hangzhou Women’s Hospital between 2020 and 2024. The study protocol was approved by the Ethics Committee of Hangzhou Women’s Hospital (Approval No. A (6)-03, 2022).

### Study population

A total of 628 women were enrolled in this study. The diagnosis of PCOS was defined according to the Rotterdam Consensus proposed in 2003. Eligible cases were required to exhibit at least two of the following three clinical features: (1) Oligo-ovulation or anovulation, (2) Biochemical or clinical hyperandrogenism, and (3) Polycystic ovarian morphology on ultrasound.

Stringent exclusion criteria were applied to ensure sample homogeneity. Women were excluded if they were aged <20 or >40 years, or had used sex hormones or any medications known to interfere with the hormonal assays within the preceding three months. Furthermore, patients with other etiologies of hyperandrogenism and ovulatory dysfunction were excluded, such as those with congenital adrenal hyperplasia, 21-hydroxylase deficiency, androgen-secreting tumors, Cushing’s syndrome, thyroid disorders, and hyperprolactinemia. Individuals with acute or chronic infections, systemic inflammatory diseases, or those with incomplete clinical data were also excluded. The control group consisted exclusively of women who did not meet any of the Rotterdam criteria. These women were healthy patients who attended the same hospital during the same period for routine physical examinations or contraceptive counseling. None had a known history of infertility, endometriosis, recurrent pregnancy loss, or other conditions known to affect ovarian reserve or AMH levels.

### Data collection

Data required for model construction were extracted from the Hospital Information System (HIS) and Laboratory Information System (LIS). The extracted data encompassed detailed patient histories, overall health status, family history, past and current medication use, and demographic details (age and body mass index, BMI) recorded during the clinical visits.

Endocrine parameters, measured during the early follicular phase (days 2–5 of the menstrual cycle), included: follicle-stimulating hormone (FSH), LH, estradiol (E2), progesterone (P), prolactin (PRL), total testosterone (T), the LH/FSH ratio, free triiodothyronine (FT3), free thyroxine (FT4), thyroid-stimulating hormone (TSH), AMH, and dehydroepiandrosterone sulfate (DHEA-S). Metabolic parameters comprised: Fasting plasma glucose (FPG), insulin, triglycerides (TG), total cholesterol (TC), low-density lipoprotein cholesterol (LDL-C), high-density lipoprotein cholesterol (HDL-C), the homeostasis model assessment of insulin resistance (HOMA-IR), and the TyG index.

All laboratory procedures adhered to international standards for medical laboratories to ensure the accuracy and reliability of results. Reproductive hormone assays were performed using a Beckman Coulter DXI800 electrochemiluminescence analyzer. AMH levels were specifically measured using a Roche Cobas e411 electrochemiluminescence analyzer. All assays utilized the manufacturer’s original, instrument-matched reagents. To ensure data accuracy and enable comprehensive analysis, all extracted information was linked for each patient via a unique health identification number.

### Measurements and definitions

1. TyG index = ln [fasting triglycerides (mg/dL) × fasting plasma glucose (mg/dL)/2].
2. HOMA-IR = [fasting insulin (mU/L) × fasting glucose (mg/dL)]/405.
3. LH/FSH ratio= luteinizing hormone/follicle stimulating hormone.

### Statistical analysis

Categorical variables were represented as frequencies and percentages, and continuous variables were represented as mean ± standard deviation (SD) ( $\bar{X}\pm SD$) or median (Q25, Q75). Group comparisons were conducted via Student’s t test for normally distributed variables and the Mann-Whitney U test for nonnormally distributed variables. Categorical variables were presented as proportions and were compared between groups via the Pearson χ² test or Fisher’s exact test. The primary analyses were performed on complete-case data (participants with no missing values for any variable), without imputation. To assess robustness, we performed a sensitivity analysis using MICE with 5 imputed datasets (26), results were consistent with the primary analysis (**Supplementary Table 1**). A two-sided *P* value of < 0.05 was considered statistically significant. All analyses were performed using the Deepwise & Beckman Coulter DxAI platform (https://www.xsmartanalysis.com). This platform was used exclusively for data management, statistical modeling (including logistic regression, restricted cubic spline analysis, and ROC curve analysis), and figure generation.

The potential nonlinear relationships of AMH and the TyG index with PCOS were further assessed using restricted cubic spline (RCS) curves based on multivariate logistic regression models (27). The Akaike information criterion (AIC) was used to determine the optimal number of knots for the RCS models by comparing models with 3 to 7 knots (**Supplementary Table 2**), with lower AIC values indicating better model fit. Ultimately, four knots were selected, positioned at the 5th, 35th, 65th, and 95th percentiles of the distribution for each variable.

Variance inflation factors (VIF) were calculated for all continuous candidate variables. Variables with VIF≥5 were considered highly collinear and were excluded from multivariable models. The remaining variables with VIF<5 were retained for further analysis.

Univariate logistic regression analyses were performed for each variable, adjusted for age and BMI. Variables with *P* < 0.10 in univariate analysis were considered candidates for further multivariable analysis.

To evaluate the independent associations of various factors with PCOS and the synergistic effect between AMH and the TyG index, binary logistic regression analyses were performed after adjusting for the confounding effects of age and BMI. These analyses included interaction term analysis, subgroup analysis, and combined pattern analysis. For subgroup and joint stratification analyses, we first determined the cut-off values for AMH and the TyG index using ROC curve analysis with the Youden index. To explore whether this synergistic effect persists in non-obese individuals, we then performed a subgroup analysis in participants with BMI < 24 kg/m² (normal-weight/lean according to Asian criteria), applying the same multivariable logistic regression model (including AMH, TyG, AMH × TyG, age, and BMI). The clinical utility of the models was evaluated using receiver operating characteristic (ROC) curves, the area under the curve (AUC), and decision curve analysis (DCA).

## Results

### Characteristics of the study population

This study ultimately included a total of 628 women (PCOS, n = 440; non-PCOS, n = 188). The main exposure variables (AMH, triglycerides, and fasting glucose) had no missing data (0.0%), and other covariates had only minimal missing rates (all < 2% except DHEA−S at 17.4%, which was not a primary exposure). Thus, missing data did not affect the primary results. As detailed in **Table 1**, compared to the control group, patients with PCOS were significantly younger (27.0 years vs. 30.0 years, *P* < 0.001) and had a higher BMI (25.39 kg/m² vs. 22.06 kg/m², *P* < 0.001).

**Table 1:** Baseline characteristics of the overall cohort. .

Characteristics	Missing rate (%)	Total (n=628)	Non-PCOS (n=188)	PCOS (n=440)	*P-value*
Age *(Year)*	0.0	28.0 [25.0, 31.0]	30.0 [26.0,33.0]	27.0 [25.0, 30.0]	<0.001
BMI *(Kg/m^2^)*	0.0	24.39 [21.09, 28.44]	22.06 [19.84, 25.95]	25.39 [22.03, 29.34]	<0.001
AMH *(ng/ml)*	0.0	5.24 [3.75, 7.23]	4.14 [2.10, 6.00]	5.69 [4.25, 7.64]	<0.001
TyG	0.0	8.33 ± 0.43	8.26 ± 0.44	8.37 ± 0.42	0.005
FPG *(mmol/L)*	0.0	4.90 [4.61, 5.21]	4.94 [4.62, 5.23]	4.88 [4.59, 5.18]	0.270
INS *(uIU/ml)*	0.0	8.20 [4.90, 13.60]	5.30 [4.40, 7.70]	10.10 [5.90, 16.80]	<0.001
HOMA-IR	0.2	1.83 [1.09, 3.03]	1.20 [0.93, 1.82]	2.19 [1.29, 3.73]	<0.001
TC *(mmol/L)*	0.0	4.74 [4.19, 5.36]	4.69 [4.16, 5.43]	4.76 [4.20, 5.34]	0.995
TG *(mmol/L)*	0.0	1.06 [0.83, 1.41]	0.99 [0.71, 1.38]	1.12 [0.86, 1.44]	0.003
HDL-C *(mmol/L)*	0.0	1.37 [1.19, 1.60]	1.43 [1.24, 1.68]	1.35 [1.16, 1.56]	0.002
LDL-C *(mmol/L)*	0.0	2.54 [2.18, 2.97]	2.45 [2.14, 2.99]	2.57 [2.18, 2.95]	0.353
FT3 *(nmol/L)*	1.6	4.42 [4.10, 4.91]	4.30 [3.99, 4.70]	4.45 [4.15, 4.97]	<0.001
FT4 *(pmol/L)*	1.6	12.91 [12.00, 13.99]	13.11 [12.00, 13.97]	12.84 [12.03, 14.00]	0.336
TSH *(mIU/L)*	1.6	1.81 [1.27, 2.56]	1.64 [1.25, 2.30]	1.85 [1.29, 2.64]	0.020
FSH *(IU/L)*	0.0	6.09 [4.64, 7.18]	6.24 [5.00, 7.54]	6.05 [4.62, 7.11]	0.051
LH *(IU/L)*	0.0	7.66 [4.90, 13.68]	5.91 [3.82, 9.77]	9.34 [5.32, 14.12]	<0.001
PRL *(ng/ml)*	0.0	12.49 [8.88, 16.31]	11.80 [8.72, 15.54]	13.24 [8.99, 16.84]	0.037
E2 *(pg/ml)*	0.0	36.98 [28.15, 55.18]	34.62 [25.95, 56.45]	38.26 [28.84, 53.94]	0.063
P *(ng/ml)*	0.0	0.58 [0.36, 1.02]	0.41 [0.34, 0.69]	0.68 [0.40, 1.19]	<0.001
T *(ng/ml)*	0.0	0.60 [0.46, 0.76]	0.49 [0.37, 0.62]	0.67 [0.51, 0.82]	<0.001
DHEA-S *(ug/dl)*	17.4	253.00 [194.00,340.00]	210.00 [154.00,262.00]	262.00 [203.00, 347.00]	<0.001
LH/FSH ratio	0.0	1.45 [0.86, 2.30]	0.98 [0.61, 1.73]	1.69 [1.08, 2.32]	<0.001
AMH quartile ^a^ *(n, %)*	0.0				
Q1		155 (24.68)	82 (43.62)	73 (16.59)	<0.001
Q2		159 (25.32)	45 (23.94)	114 (25.91)	
Q3		157 (25.00)	32 (17.02)	125 (28.41)	
Q4		157 (25.00)	29 (15.42)	128 (29.09)	

Data presented are mean ± SD, median (IQR), or n (%).

BMI, Body mass index; PCOS, Polycystic ovary syndrome; AMH, Anti-Müllerian Hormone; TyG, Triglyceride-Glucose index; FPG, Fasting plasma glucose; INS, Insulin; HOMA-IR, Homeostasis model assessment of insulin resistance; TC, Total cholesterol; TG, Triglycerides; HDL-C, High-density lipoprotein cholesterol; LDL-C, Low-density lipoprotein-C; FT_3_, Free triiodothyronine; FT_4_, Free thyroxine; TSH, Thyroid stimulating hormone; FSH, Follicle stimulating hormone; LH, Luteinizing hormone; PRL, Prolactin; E_2_, Estradiol; P, Progesterone; T, Testosterone; DHEA-S, Dehydroepiandrosterone sulfate.

a, AMH quartile Q1: < 3.75; Q2: 3.75 – 5.22; Q3: 5.23 – 7.24; Q4: ≥ 7.25.

Regarding reproductive endocrine profiles, serum levels of AMH, LH, P, T, DHEA-S, and the LH/FSH ratio were all significantly elevated in the PCOS group compared to the controls (all *P* < 0.001). PRL levels were also modestly higher in the PCOS group (*P* < 0.05). Stratification by AMH quartiles revealed a significant positive trend, with the proportion of PCOS cases increasing markedly across higher AMH quartiles (trend *P* < 0.001).

In terms of metabolic parameters, the PCOS group exhibited significantly higher values for the TyG index, INS, HOMA-IR, and TG levels compared to the control group (all *P* < 0.05), while HDL-C levels were significantly lower (*P* = 0.002). Additionally, FT_3_ and TSH levels were slightly higher in PCOS patients (*P* < 0.05). However, no statistically significant differences were observed between the two groups for FPG, TC, LDL-C, FT_4_, FSH, or E_2_ levels.

### Multicollinearity assessment

Variance inflation factors (VIF) were calculated for all continuous candidate variables. After excluding highly collinear variables (e.g., INS, triglycerides, total testosterone, LH/FSH ratio) that had VIF ≥5, all remaining variables had VIF <5 (range 1.089–2.020; **Supplementary Table 3**), indicating no significant multicollinearity.

### Dose−response relationships of AMH and TyG index with PCOS risk

**Figure 1:**
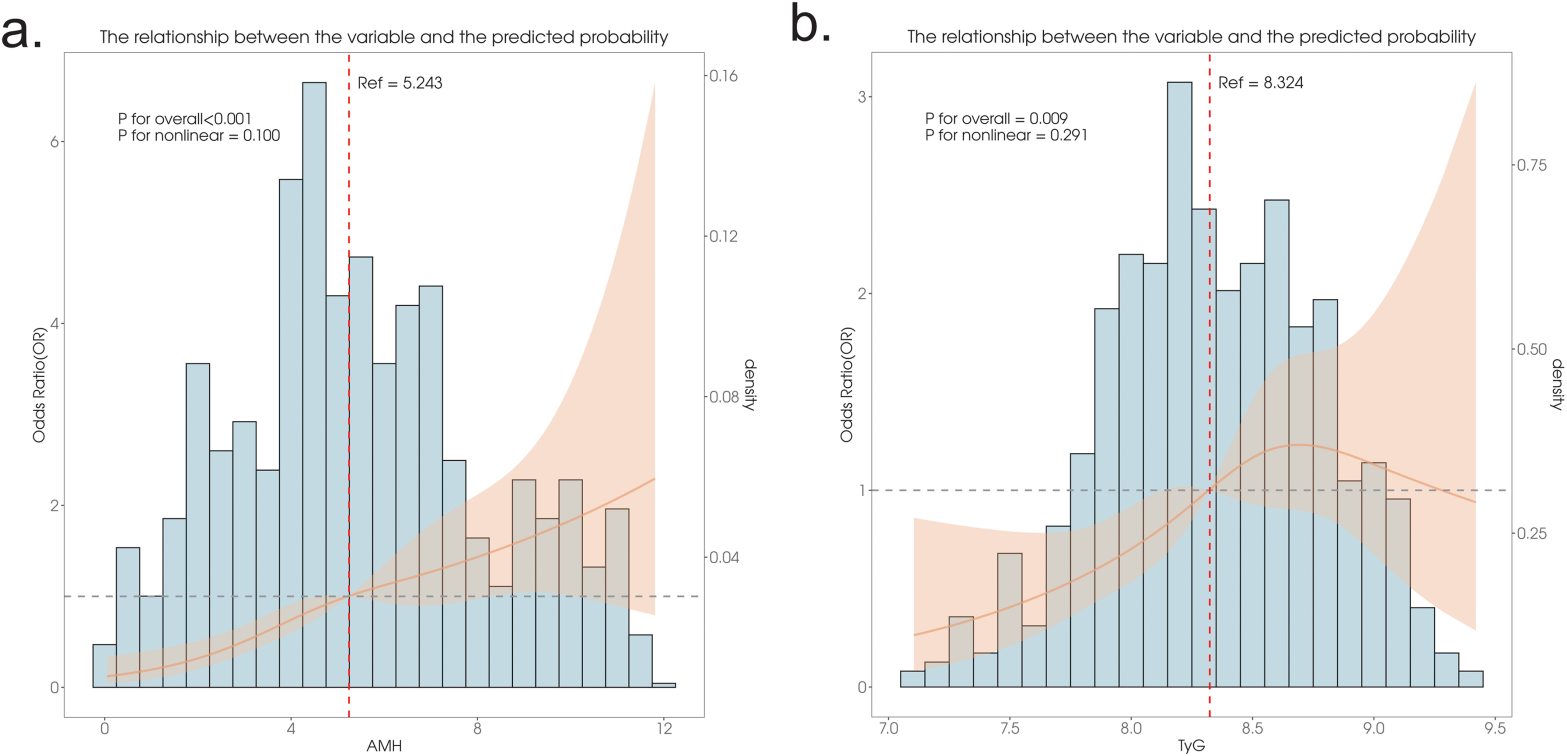
RCS curves for **(a)** AMH and **(b)** TyG index with PCOS risk, adjusted for age and BMI, with 4 knots placed at the 5th, 35th, 65th, and 95th percentiles.

Restricted cubic spline regression with four knots was used to assess potential nonlinear associations after adjusting for age and BMI. As shown in **Figure 1a**, AMH demonstrated a linear positive association with PCOS risk (*P* for nonlinearity = 0.100). The odds ratio for PCOS increased progressively with AMH levels above the reference value of 5.243 ng/mL, whereas lower AMH levels were associated with a slightly decreased risk. The TyG index also exhibited a linear positive association with PCOS risk (*P* for nonlinearity = 0.291; **Figure 1b**), with the odds ratio rising steadily across the observed TyG range.

### Independent and interactive effects of AMH and the TyG index on PCOS

To quantify the independent and combined effects of AMH and the TyG index on the risk of PCOS. As detailed in **Table 2**, logistic regression analysis was performed. The results demonstrated that in the unadjusted model (Model 1), both AMH and the TyG index were significantly associated with an increased risk of PCOS (AMH, OR = 1.286, 95% CI: 1.196–1.383, *P* < 0.001; TyG index, OR = 1.559, 95% CI: 1.045–2.325, *P* = 0.029). The AMH & TyG also showed a significant positive association (OR = 1.032, 95% CI: 1.023–1.041, *P* < 0.001). After adjusting for potential confounders including age and BMI (Model 2), these associations remained statistically significant. Specifically, the adjusted OR for AMH was 1.252 (95% CI: 1.153–1.359, *P* < 0.001). The OR for the TyG index increased to 1.981 (95% CI: 1.267–3.099, *P* = 0.003), and the combined AMH & TyG indicator remained a significant predictor, with an adjusted OR of 1.029 (95% CI: 1.019–1.039, *P* < 0.001).

**Table 2:** ORs (95% CIs) for PCOS according to the AMH and TyG.

Categories	Model 1 OR 95%CI	*P-value*	Model 2 OR 95%CI	*P-value*
AMH	1.286 [1.196,1.383]	<0.001	1.252 [1.153,1.359]	<0.001
TyG	1.559 [1.045,2.325]	0.029	1.981 [1.267,3.099]	0.003
AMH & TyG	1.032 [1.023,1.041]	<0.001	1.029 [1.019 1.039]	<0.001

OR, Odds ratio; CI, Confidence interval; PCOS, Polycystic ovary syndrome; AMH, Anti-Müllerian Hormone; TyG, Triglyceride-glucose index.

Model 1, Unadjusted.

Model 2, Adjusted for age; body mass index.

### Subgroup analysis in normal-weight/lean participants

To address the concern that the TyG index may not capture insulin resistance in lean individuals, we repeated the above multivariable logistic regression model in participants with BMI < 24 kg/m² (n = 291, 46.3% of the total cohort). As shown in **Supplementary Table 4**, the synergistic interaction between AMH and TyG remained significant after adjusting for age and BMI (adjusted OR for interaction = 1.033, 95% CI: 1.020–1.046, *P* < 0.001). Both AMH (adjusted OR = 1.280, 95% CI: 1.151–1.422, *P* < 0.001) and TyG (adjusted OR = 2.803, 95% CI: 1.530–5.133, *P* = 0.003) were also independently associated with PCOS in this subgroup. These findings indicate that the metabolic-reproductive synergy is not driven by obesity and is present even in normal-weight/lean PCOS patients.

### Univariate logistic regression associations of individual biomarkers with PCOS

**Figure 2:**
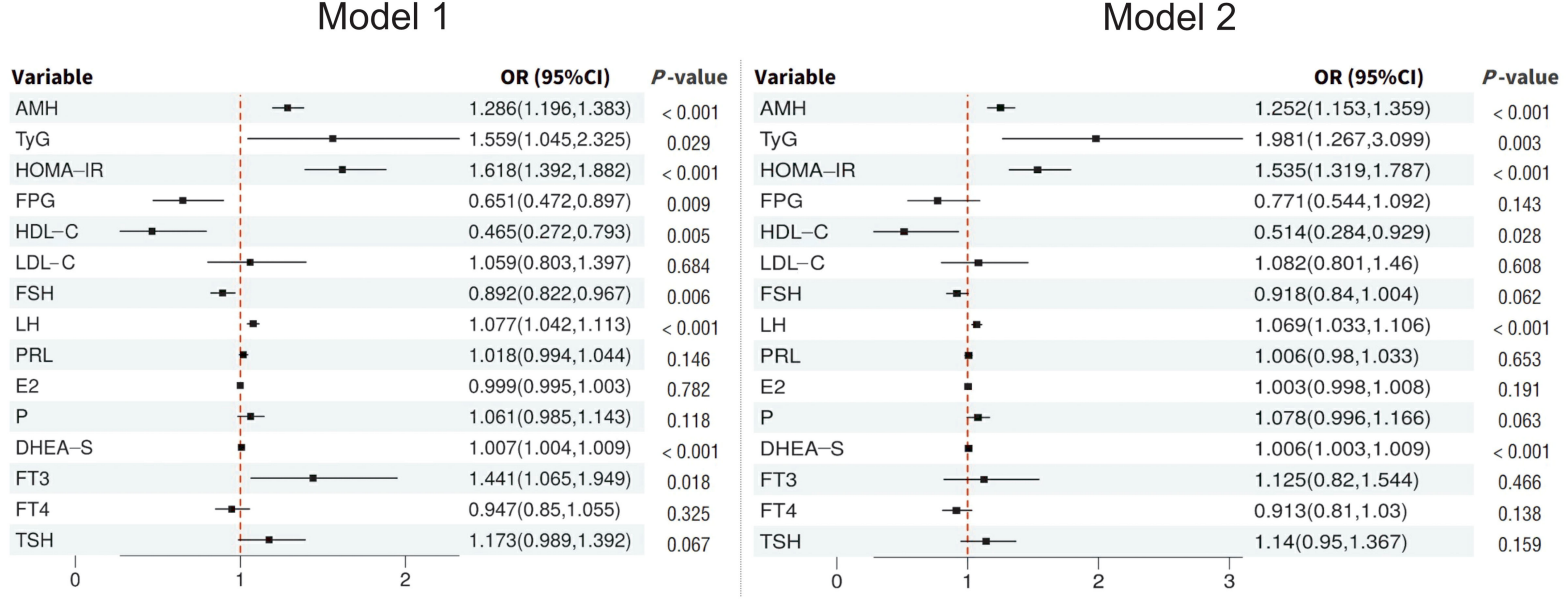
Univariate logistic regression associations of individual biomarkers with PCOS. Model 1: Unadjusted; Model 2: Adjusted for age; body mass index.

After excluding highly collinear variables (VIF ≥ 5), only biomarkers with VIF < 5 were retained for analysis, as shown in **Figure 2**. The detailed associations for AMH and the TyG index are presented above. Logistic regression analysis revealed that in the unadjusted model (Model 1), multiple biomarkers were significantly associated with PCOS risk. Among them, AMH (OR = 1.286, *P* < 0.001) and the TyG index (OR = 1.559, *P* = 0.029) demonstrated strong associations. After adjusting for age and BMI (Model 2), the associations for AMH (OR = 1.252, *P* < 0.001) and the TyG index (OR = 1.981, *P* = 0.003) remained highly significant, with the strength of the association for the TyG index being notably enhanced. Furthermore, the homeostasis model assessment of HOMA-IR, HDL-C, LH, and DHEA-S also remained significant independent risk factors for PCOS (all *P* < 0.05).

### Examining the TyG index-PCOS association across AMH strata

Subgroup analysis revealed that AMH levels significantly modified the association between the TyG index and PCOS risk. In the low AMH subgroup (≤3.85 ng/mL), the TyG index showed a strong positive correlation with PCOS risk. This association remained highly significant both in the unadjusted model (OR = 3.184, 95% CI: 1.468–6.905, *P* = 0.003) and after adjustment for age and BMI (OR = 4.561, 95% CI: 1.779–11.698, *P* = 0.002). In contrast, in the high AMH subgroup (>3.85 ng/mL), this association was markedly attenuated and became statistically non-significant (adjusted OR = 1.490, 95% CI: 0.858–2.588, *P* = 0.157) (**Table 3**).

**Table 3:** Stratified analysis of the TyG index–PCOS association by AMH level.

Categories	Low AMH Subgroup (≤3.85 *ng/ml*)		High AMH Subgroup (>3.85 *ng/ml*)	
	OR 95%CI	*P-value*	OR 95%CI	*P-value*
TyG ^a^	3.184 [1.468, 6.905]	0.003	1.362 [0.790, 2.227]	0.286
TyG ^b^	4.561 [1.779, 11.698]	0.002	1.490 [0.858, 2.588]	0.157

OR, Odds ratio; CI, Confidence interval; PCOS, Polycystic ovary syndrome; AMH, Anti-Müllerian Hormone; TyG, Triglyceride-Glucose index.

a, Unadjusted.

b, Adjusted for age, body mass index.

### Risk stratification by combined AMH and TyG categories

As shown in **Table 4**, a clear gradient of increasing PCOS risk was observed across the four groups. After adjustment for age and BMI, this risk gradient persisted and remained statistically significant. The adjusted ORs were 3.582 (95% CI: 1.506–8.517, *P* = 0.004) for the Low AMH & High TyG group, 5.894 (95% CI: 2.508–13.850, *P* < 0.001) for the High AMH & Low TyG group, and 12.082 (95% CI: 5.350–27.284, *P* < 0.001) for the High AMH & High TyG group.

**Table 4:** PCOS risk stratification by AMH and TyG levels.

Categories	Model 1 OR 95%CI	*P-value*	Model 2 OR 95%CI	*P-value*
Low AMH & Low TyG	*Ref.*		*Ref.*	
Low AMH & High TyG	3.474 [1.610, 7.494]	0.002	3.582 [1.506, 8.517]	0.004
High AMH & Low TyG	8.053 [3.701, 17.521]	<0.001	5.894 [2.508, 13.850]	<0.001
High AMH & High TyG	13.050 [6.240, 27.292]	<0.001	12.082 [5.350, 27.284]	<0.001

OR, Odds ratio; CI, Confidence interval; PCOS, Polycystic ovary syndrome; AMH, Anti-Müllerian Hormone; TyG, Triglyceride-Glucose index; Ref., Reference group.

Model 1, Unadjusted.

Model 2, Adjusted for age; body mass index.

Low AMH ≤ 3.85, High AMH>3.85; Low TyG ≤ 8.11, High TyG>8.11.

### Comparison of the effectiveness of AMH, TyG, and combined models for predicting PCOS risk

**Figure 3:**
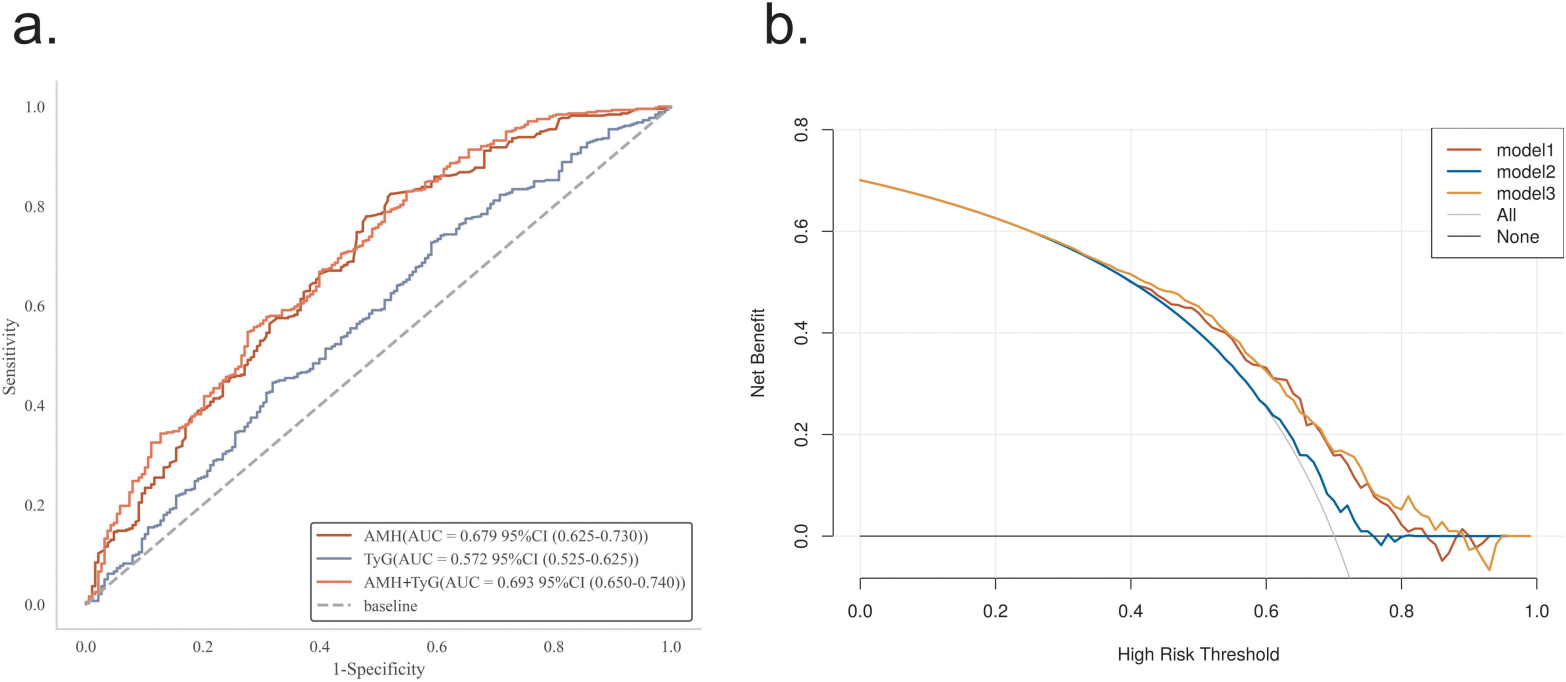
**(a)** ROC for the prediction of PCOS; **(b)** DCA for PCOS prediction: Model 1 (AMH alone), Model 2 (TyG index alone), and Model 3 (combined AMH and TyG index).

ROC curve analysis was performed to evaluate the models’ discriminative ability. The results indicated that the combined model incorporating both AMH and the TyG index (AMH & TyG) achieved the highest predictive performance, with an area under the curve (AUC) of 0.693. This was slightly higher than the AUC of the AMH-alone model (AUC = 0.679) and notably higher than that of the TyG-alone model (AUC = 0.572) (**Figure 3a**). Furthermore, DCA was conducted to explore the net benefit of the models across various decision thresholds. The analysis suggested that both the AMH-alone model and the combined AMH & TyG model showed higher net benefit compared to the TyG-alone model within certain threshold ranges, indicating their potential exploratory utility (**Figure 3b**).

## Discussion

In this retrospective cross-sectional study of 628 women, we demonstrated associations between AMH, TyG index, and the risk of PCOS. The results indicated that higher levels of AMH and TyG were independently associated with an increased risk of PCOS, even after adjusting for key confounders such as age and BMI. The RCS curves showed that both AMH and the TyG index had a positive linear association with PCOS risk; AMH and the TyG index exhibited significant synergistic effects in risk assessment, and their combined use provided superior predictive performance for PCOS risk compared to either marker alone. In summary, our findings suggest that AMH and the TyG index may be useful for characterizing PCOS risk in research settings, but external validation is required before any clinical application. The combined assessment warrants further investigation in prospective, multi-center cohorts.

Consistent with previous studies (24, 28–30), our data indicate that patients with PCOS exhibit a distinct profile characterized by younger age, higher BMI, and significantly elevated levels of AMH, LH, T, and the TyG index compared to controls. Identifying key risk and prognostic factors in this population is therefore crucial for improving clinical management. AMH, an important biomarker reflecting ovarian reserve and antral follicle count (31), has been widely documented to be significantly elevated in women with PCOS (15). The 2023 international evidence-based guideline further supports the use of AMH levels as a substitute for ovarian ultrasound in adults (11). Moreover, the linear positive correlation observed between AMH and PCOS risk in our study reinforces the role of AMH as a core biomarker of ovarian pathology in PCOS, which aligns with prior research recognizing it as a hallmark indicator of the disorder (32). Similarly, the TyG index, a composite measure derived from fasting TG and FPG (23, 33), has been widely validated as a reliable surrogate biomarker for assessing IR, owing to its favorable sensitivity and specificity (34) along with its clinical practicality and cost-effectiveness (35). Previous research has indicated that an elevated baseline TyG index or excessive exposure is associated with an increased risk of PCOS (24). In the present study, although the nonlinear relationship between the TyG index and PCOS risk did not reach statistical significance, its trend was consistent with the linear positive association identified in the logistic regression analysis. Together, these findings suggest that metabolic deterioration constitutes a persistent risk factor for PCOS. This observation provides epidemiological support for the central role of insulin resistance in the pathogenesis of PCOS, wherein IR exacerbates hyperandrogenemia by stimulating ovarian androgen synthesis and reducing sex hormone-binding globulin levels, while also directly contributing to a spectrum of metabolic complications, including dyslipidemia and glucose metabolism disorders. Therefore, the significant synergistic interaction between AMH and the TyG index, along with their central role in multivariable models, provides crucial insight for understanding the inherent high heterogeneity of PCOS.

Importantly, the synergistic interaction between AMH and TyG remained significant in the subgroup of normal-weight/lean participants (BMI < 24 kg/m²), suggesting that the TyG index, despite being a triglyceride-based marker, still captures clinically meaningful insulin resistance-related risk even in the absence of obesity. This finding suggests that functional insulin resistance in lean PCOS patients may not be reflected by lipid-based indices.

Notably, subgroup analysis revealed a nuanced dynamic relationship between the TyG index, AMH levels, and the risk of PCOS. The strength of the association between the TyG index and PCOS varied across AMH strata, with a particularly pronounced effect observed in the low AMH subgroup. This suggests that this population may align with a clinical phenotype characterized predominantly by “metabolic disturbance”. Within this phenotype, IR is not merely a common feature but may act as a key initiator or perpetuating factor in the development of hyperandrogenism and ovulatory dysfunction, thereby playing a central role in the pathogenesis and progression of PCOS (36, 37). In contrast, among women with significantly elevated AMH levels, the pathogenesis of PCOS appears to stem more from intrinsic ovarian dysfunction. High AMH levels can directly disrupt follicular development and selection by inhibiting aromatase activity and reducing follicular sensitivity to FSH, leading to a phenotype dominated by “ovarian hyper-responsiveness” or “folliculogenic” abnormality (38). In this group, although insulin resistance is frequently detected, its independent association with PCOS risk is relatively weaker, implying that insulin resistance may primarily serve a modifying or interactive role on top of pre-existing ovarian dysfunction. This finding further substantiates the theoretical perspective that PCOS represents a syndrome of marked heterogeneity.

Furthermore, the joint risk stratification based on AMH and TyG levels reveals the complexity of PCOS risk. Interestingly, in this study we observed that compared to the low AMH/low TyG group, the high AMH/high TyG group exhibited the highest risk of PCOS, which was significantly greater than that of groups with only one elevated indicator, suggesting a clear synergistic effect between metabolic abnormalities and ovarian hyperresponsiveness. This finding supports our core scientific hypothesis: PCOS is not a disease driven by a single mechanism, but rather a heterogeneous syndrome resulting from the varying degrees of overlap or interaction between metabolic disorders and ovarian functional abnormalities.

To assess the predictive performance, ROC and DCA were employed for AMH, TyG, and their combination. The combined model integrating both metabolic and reproductive dimensions showed modest discrimination (AUC = 0.693) compared with either marker alone. DCA also suggested potential net benefit across certain threshold probabilities, though these findings are exploratory and require validation.

Compared with previous studies that primarily focused on single dimensions (8, 39, 40), the innovation of this research lies in the integration of both reproductive endocrine (AMH) and metabolic (TyG index) indicators, and in the systematic quantification of their interaction in relation to PCOS risk. Based on the above analysis, we hypothesize that combined assessment of AMH and the TyG index may help characterize PCOS risk into distinct profiles. For example, patients with markedly elevated AMH but relatively normal metabolic parameters might benefit from clinical focus on ovarian function and fertility preservation, whereas those with normal or low AMH but significantly elevated TyG index might prioritize improving insulin sensitivity and addressing metabolic disturbances. These hypotheses are preliminary and require prospective validation before any clinical application.

It is worth discussing that, even though we did not perform phenotype-stratified analyses, how to interpret the value of AMH and the TyG index across different PCOS phenotypes remains an important question. The four PCOS phenotypes defined by the Rotterdam classification differ considerably in metabolic and reproductive characteristics. Phenotype A (hyperandrogenism + oligo-anovulation + polycystic ovarian morphology) and phenotype B (hyperandrogenism + oligo-anovulation) are characterized by more severe insulin resistance and hyperandrogenism, whereas phenotype C (hyperandrogenism + polycystic ovarian morphology) shows an intermediate degree of metabolic abnormality, and phenotype D (oligo-anovulation + polycystic ovarian morphology) exhibits the mildest metabolic abnormalities (3, 41). Given that the TyG index reflects insulin resistance, it may be significantly elevated in phenotypes A and B and could have clinical utility (24). In contrast, AMH, as a marker of ovarian reserve, is also present at higher levels in phenotype D, a non-hyperandrogenic PCOS phenotype (42). Therefore, we speculate that the synergistic effect between AMH and the TyG index observed in our study might be driven mainly by phenotypes A and B, whereas AMH may play a dominant role in phenotype D. This hypothesis warrants validation in future phenotype-stratified studies to clarify the performance of these markers across different PCOS phenotypes.

## Limitations

This study had limitations. First, the cross-sectional design cannot establish causal relationships. Second, as a single-center retrospective analysis, the relatively homogeneous sample source may introduce hospital-based selection bias, potentially limiting the generalizability of the conclusions. Third, constrained by resources, the study did not perform subtype analysis of PCOS (including Rotterdam phenotypes A–D) nor include other potential risk factors such as lifestyle and genetic factors. Therefore, we could not investigate the correspondence between the combined AMH/TyG categories and specific clinical subtypes. This represents an important direction for our future research. Fourth, the combined model achieved only modest discriminative ability, and no internal (e.g., cross-validation) or external validation was performed; therefore, our findings are hypothesis-generating and should not be interpreted as supporting clinical risk stratification or precision medicine applications. Fifth, although we excluded controls with a known history of infertility, endometriosis, or recurrent pregnancy loss, we cannot completely rule out the presence of undiagnosed subclinical reproductive or endocrine conditions (e.g., subclinical PCOS, thyroid dysfunction, or hyperprolactinemia) that might theoretically affect AMH levels. However, the normal AMH, BMI, HOMA-IR, and FPG values in the control group suggest that the impact of such potential bias is likely limited. Future research should employ prospective, multi-center, large-sample cohort studies to validate the stability and generalizability of the proposed model.

## Conclusions

In summary, combined assessment of AMH and the TyG index showed marginally improved predictive performance for PCOS in this single-center study. Further validation in prospective cohorts is required before clinical application.

## Data Availability

The datasets used and/or analyzed during the current study are available from the co-first author on reasonable request. Requests to access the datasets should be directed to YC, 64684154@qq.com.
