# Supplementary material for: Combined metabolic-reproductive association and predictive value of AMH and TyG index in PCOS: a single-center retrospective study
Source: Front Endocrinol (Lausanne). 2026 Jul 8;17:1847801. doi: 10.3389/fendo.2026.1847801 (PMC13388220; doi:10.3389/fendo.2026.1847801)
Supplement: Supplementary file 1 [file Table1.docx]

**Supplementary Table S1.** Comparison of results between complete‑case and multiple imputation analyses.

| **Variable** | **Complete‑case analysis**  OR (95% CI) | *P*-value | **MICE analysis**  OR (95% CI) | *P*-value |
| --- | --- | --- | --- | --- |
| AMH | 1.252 [1.153, 1.359] | <0.001 | 1.252 [1.153,1.359] | <0.001 |
| TyG | 1.981 [1.267, 3.099] | 0.003 | 1.981 [1.267,3.099] | 0.003 |
| HOMA-IR | 1.535 [1.319, 1.787] | <0.001 | 1.529 [1.315,1.778] | <0.001 |
| FPG | 0.771 [0.544, 1.092] | 0.143 | 0.771 [0.544,1.092] | 0.143 |
| HDL-C | 0.514 [0.284, 0.929] | 0.028 | 0.514 [0.284,0.929] | 0.028 |
| LDL-C | 1.082 [0.801, 1.460] | 0.608 | 1.082 [0.801,1.460] | 0.608 |
| FSH | 0.918 [0.840, 1.004] | 0.062 | 0.918 [0.840,1.004] | 0.062 |
| LH | 1.069 [1.033, 1.106] | <0.001 | 1.069 [1.033,1.106] | <0.001 |
| PRL | 1.006 [0.980, 1.033] | 0.653 | 1.006 [0.980,1.033] | 0.653 |
| E2 | 1.003 [0.998, 1.008] | 0.191 | 1.003 [0.998,1.008] | 0.191 |
| P | 1.078 [0.996, 1.166] | 0.063 | 1.078 [0.996,1.166] | 0.063 |
| DHEA-S | 1.006 [1.003, 1.009] | <0.001 | 1.003 [1.000,1.005] | 0.023 |
| FT3 | 1.125 [0.820, 1.544] | 0.466 | 1.144 [0.835,1.566] | 0.403 |
| FT4 | 0.913 [0.810, 1.030] | 0.138 | 0.919 [0.816,1.035] | 0.162 |
| TSH | 1.140 [0.950, 1.367] | 0.159 | 1.135 [0.949,1.357] | 0.166 |

**Notes:**Adjusted for age and BMI. Complete‑case analysis was performed on participants with complete data for all variables (n = 628). MICE analysis was performed with 5 imputed datasets. Results were consistent between the two approaches, supporting the robustness of the findings.

**Abbreviations:** ***AMH*** Anti-Müllerian Hormone, ***TyG*** Triglyceride-Glucose index, ***FPG*** Fasting plasma glucose, ***HOMA-IR*** Homeostasis model assessment of insulin resistance, ***HDL-C*** High-density lipoprotein cholesterol, ***LDL-C*** Low-density lipoprotein-C, ***FSH*** Follicle stimulating hormone, ***LH*** Luteinizing hormone, ***PRL*** Prolactin, ***E_2_*** Estrogen, ***P*** Progesterone, ***DHEA-S*** Dehydroepiandrosterone sulfate, ***FT_3_*** Free triiodothyronine, ***FT_4_*** Free thyroxin, ***TSH*** Thyroid stimulating hormone.
