# Supplementary material for: Combined metabolic-reproductive association and predictive value of AMH and TyG index in PCOS: a single-center retrospective study
Source: Front Endocrinol (Lausanne). 2026 Jul 8;17:1847801. doi: 10.3389/fendo.2026.1847801 (PMC13388220; doi:10.3389/fendo.2026.1847801)
Supplement: Supplementary file 2 [file Table2.docx]

**Supplementary Table S2.** Comparison of AIC values for RCS models with different numbers of knots, adjusted for age and BMI.

| **Variable** | **Knots** | **AIC** |
| --- | --- | --- |
| AMH | 3 | 1234.5 |
|  | 4 | 1230.1 |
|  | 5 | 1231.8 |
|  | 6 | 1233.0 |
|  | 7 | 1235.4 |
| TyG index | 3 | 1245.2 |
|  | 4 | 1242.7 |
|  | 5 | 1243.9 |
|  | 6 | 1245.1 |
|  | 7 | 1247.6 |

**Notes:** Adjusted for age and BMI; knots at 5th, 35th, 65th, and 95th percentiles. The lowest AIC for each variable is shown in bold.

**Abbreviations: *AIC*** Akaike information criterion, ***AMH*** Anti-Müllerian Hormone, ***TyG*** Triglyceride-Glucose index, ***BMI*** Body mass index
