# Supplementary material for: Combined metabolic-reproductive association and predictive value of AMH and TyG index in PCOS: a single-center retrospective study
Source: Front Endocrinol (Lausanne). 2026 Jul 8;17:1847801. doi: 10.3389/fendo.2026.1847801 (PMC13388220; doi:10.3389/fendo.2026.1847801)
Supplement: Supplementary file 3 [file Table3.docx]

**Supplementary Table S3.** VIF for continuous variables included in the final multivariable models

| **Characteristics** | **VIF** |
| --- | --- |
| P | 2.020 |
| TyG | 2.000 |
| E2 | 1.997 |
| FSH | 1.903 |
| T | 1.874 |
| DHEA-S | 1.752 |
| HOMA-IR | 1.679 |
| LH | 1.610 |
| LDL-C | 1.409 |
| Age | 1.363 |
| HDL-C | 1.338 |
| FPG | 1.333 |
| AMH | 1.269 |
| FT3 | 1.243 |
| PRL | 1.181 |
| FT4 | 1.153 |
| TSH | 1.095 |
| BMI | 1.089 |

**Notes:** VIF values <5 indicate no significant multicollinearity. Variables with VIF ≥5 (e.g., INS, TG, TC, LH/FSH ratio) were excluded from the final models.

**Abbreviations:** ***VIF*** Variance inflation factors, ***BMI*** Body mass index, ***AMH*** Anti-Müllerian Hormone, ***TyG*** Triglyceride-Glucose index, ***FPG*** Fasting plasma glucose, ***HOMA-IR*** Homeostasis model assessment of insulin resistance, ***HDL-C*** High-density lipoprotein cholesterol, ***LDL-C*** Low-density lipoprotein-C, ***FT_3_*** Free triiodothyronine, ***FT_4_*** Free thyroxin, ***TSH*** Thyroid stimulating hormone, ***FSH*** Follicle stimulating hormone, ***LH*** Luteinizing hormone, ***PRL*** Prolactin, ***E_2_*** Estrogen, ***P*** Progesterone, ***T*** Testosterone, ***DHEA-S*** Dehydroepiandrosterone sulfate.
