# Supplementary material for: Combined metabolic-reproductive association and predictive value of AMH and TyG index in PCOS: a single-center retrospective study
Source: Front Endocrinol (Lausanne). 2026 Jul 8;17:1847801. doi: 10.3389/fendo.2026.1847801 (PMC13388220; doi:10.3389/fendo.2026.1847801)
Supplement: Supplementary file 4 [file Table4.docx]

**Supplementary Table S4.** Multivariable logistic regression analysis of PCOS risk factors in normal‑weight/lean participants (BMI < 24 kg/m²)

| **Categories** | **Model 1**  **OR 95%CI** | ***P-value*** | **Model 2**  **OR 95%CI** | ***P-value*** |
| --- | --- | --- | --- | --- |
| AMH | 1.352 [1.222, 1.495] | <0.001 | 1.280 [1.151,1.422] | <0.001 |
| TyG | 1.920 [1.114, 3.309] | 0.019 | 2.803 [1.530,5.133] | 0.003 |
| AMH & TyG | 1.040 [1.027, 1.053] | <0.001 | 1.033 [1.020, 1.046] | <0.001 |

***OR*** Odds ratio, ***CI*** Confidence interval, ***PCOS*** Polycystic ovary syndrome, ***AMH*** Anti-Müllerian Hormone, ***TyG*** Triglyceride-glucose index

Model 1: Unadjusted

Model 2: Adjusted for age; body mass index.
